# 

**Published:** 1873-02

**Authors:** 


### *Married.*

On Jan. 14th, at Hillside, the residence of John T. Graham, Mt. Washington, Md., by Rev. R. H. Clarkson, Bishop of Nebraska, Dr. P. H. ELLSWORTH, of Hot Springs, Ark., to SARAH E., eldest daughter of Dr. C. H. Van Patten, of San Jose, Costa Rica, formerly of Washington, D. C. No cards.

The Editors congratulate.

